# Attention or Distraction? The Impact of Mobile Phone on Users' Psychological Well-Being

**DOI:** 10.3389/fpsyg.2021.612127

**Published:** 2021-04-20

**Authors:** Jianxun Chu, Sara Qaisar, Zakir Shah, Afsheen Jalil

**Affiliations:** ^1^Department of Science and Technology Communication and Policy, University of Science and Technology of China, Hefei, China; ^2^College of Media and International Culture, Zhejiang University, Hangzhou, China; ^3^Department of Technology Management, International Islamic University, Islamabad, Pakistan

**Keywords:** mobile phone, distraction, attention control, cognitive emotional pre-occupation, psychological well-being

## Abstract

Cumulative evidence has demonstrated that mobile phone distraction, in particular among emerging adults, is a growing problem. Considerable efforts have been made to contribute to the literature by proposing cognitive emotion pre-occupation which acts as an underlying mechanism through which mobile phone distraction results in a reduction in psychological well-being. The proposed model is supported by distraction-conflict theory which reveals that users, with high attention control, are better at coping with the negative consequences of mobile phone distraction. The data, consisting of 914 University students in China, was analyzed using statistical tools. The results support that mobile phone distraction has a significant positive relationship with cognitive emotional pre-occupation which negatively affects users' psychological well-being. Our findings also reveal that attention control moderated the mediation effect of cognitive emotional pre-occupation in association with mobile phone distraction and psychological well-being. The theoretical and practical implications are also discussed along with limitations and future research.

## Introduction

Mobile phone technology has become a major part of people's daily life. People, especially youths use mobile technology for various purposes (Soyemi Jumoke, 2015; Alalwan et al., 2018). Mobile phone manufacturers offer new features and functionalities that have compelled users to use them (Zheng and Lee, 2016). The versatility of the mobile phone allows seamless integration of work, fun, social interaction, and enhances the quality of life in many ways (Zhang and Adipat, 2005; David et al., 2015; Longstreet and Brooks, 2017). According to the report generated by the China Internet Network Information center in 2019, 98.6% of internet users in China had access to the internet *via* mobile devices in 2018—1.1% higher than a year earlier. People aged between 10 and 39 years accounted for 67.8% of all internet users in China, where students (25.4%) were the largest user group (CNNIC, 2019). In China, young adolescents are very fond of using a mobile phone in their daily routine activities such as during working, driving, and studying making it their first priority (Zhou, 2019), however, the negative consequences of the continuous usage of a mobile phone have been illustrated in recent studies. For example, the overuse of mobile phones has adverse effect on users' academic performance (Thomée et al., 2011; Lepp et al., 2015; Anderson et al., 2017), and work performance (Turel et al., 2011) and also cause technology driven consequences (e.g., distraction) (Coursaris et al., 2012). The problematic use of a mobile phone has become a societal debate; therefore, it is important to investigate the negative consequences of mobile phone usage in China. One of the reasons for the negative consequences of mobile phone technology is distraction (Sobhani and Farooq, 2018).

Mobile phone distraction (MD) is defined as the prevention of giving full attention to the nearest surroundings (David et al., 2015). The cognitive demand related to phone calls, email, texting, playing games, browsing, and social networking sites on mobile phones grabs user's attention or moves their attention away from other things so that they are not be able to focus on work-related activities. The mobile phone limits the user's attention and to make appropriate timely decisions and ultimately affects their psychological well-being (Salehan and Negahban, 2013). Psychological well-being is described as the overall psychological effectiveness of an individual (Gechman and Wiener, 1975; Sekaran, 1985). It measures the hedonic or pleasant aspect of individual feelings (Russell, 1980). Researchers have started analyzing the dark side of excessive mobile phone use on psychological well-being such a stress, depression, anxiety, and sleep disturbance (Bianchi and Phillips, 2005; Thomée et al., 2011; Nawaz et al., 2018). Many studies have focused on exploring the nature, measurement, and dimensions of the excessive use of technology (Chesley, 2005; Porter and Kakabadse, 2006; Thomée et al., 2007; Sahin and Çoklar, 2009; Choi and Lim, 2016). While many other research studies have investigated the cognitive and behavioral interconnections, particularly regarding negative consequences of mobile devices (Thomée et al., 2011; Turel et al., 2011; Turel and Serenko, 2012; Salehan and Negahban, 2013; Luqman et al., 2017; Cao et al., 2018; Volkmer, 2019).

Recent research studies have analyzed the impact of mobile phone distraction on social media use at work (Mark et al., 2018), during studying (David et al., 2015) and also its impact on memory and cognition (Craik, 2014). However, the negative consequences of mobile phone distraction have not been fully addressed in these previous studies. Due to an existing gap in previous research, it is important to study the negative consequences of mobile phone distraction.

This research study aims to examine how mobile phone distraction stimulates cognitive emotional pre-occupation which ultimately affect users' psychological well-being. Meanwhile, individuals' attentional control helps to enhance their psychological well-being (Ellis et al., 2014). Attention control refers to an individual's ability to focus only on those stimuli relevant to the current goal, minimizing the extent to which bottom-up influences capture our attention (Buschman and Miller, 2007). A few researchers have suggested that attention plays a critical role in reducing cognitive processing information by focusing and concentrating on the main objective (Wolfe et al., 2004; Buschman and Miller, 2007). Therefore, this study examines how attention control moderates the association between mobile phone distraction, cognitive emotional pre-occupation, and psychological well-being.

This study involves four main objectives intended to make both theoretical and practical contributions to the existing literature. First, the study examines the impact of mobile phone distraction on users' psychological well-being using distraction-conflict theory. Second, the study examines how users' cognitive emotional pre-occupation mediates the relationships between mobile phone distraction and psychological well-being. Third, the current study analyzes the moderating effect of attention control on the association between mobile phone distraction and cognitive emotional pre-occupation of users. Finally, the study examines whether attention control moderate the mediating effect of cognitive emotional pre-occupation between mobile phone distraction and psychological well-being.

## Theoretical Background and Hypothesis Development

### Distraction-Conflict Theory

According to Leung (2015), a distraction is something that makes it hard for one to think or pay attention. It is a process by which an individual or group is distracted from the desired focus area, blocking, or reducing the desired information. Robert Baron's theory of distraction-conflict based on the idea that being aware of another object creates a conflict between attending to that object and attending to the task at hand (Baron, 1986). Similarly, the distraction conflict model has three major steps (I) Others distract, (II) distraction causes attention to conflict, and (III) attention conflict elevates stress (Nicholson et al., 2005). In the presence of others, there is a conflict between the object of attention and attending to the task that causes attention conflict (Baron et al., 1978). Attention conflict refers to the situation in which the person feels a strong urge, desire, or obligation to pay attention to the distractor (i.e., mobile phone) during performing their tasks, especially when the distractor is attention-grabbing and difficult to ignore (Baron, 1986). To be able to participate in more than one stimulus at a time, a person needs greater mental activity in the working memory of an individual (Sweller, 1988, 1994), known as a cognitive load (Grieve et al., 2014). Increased cognitive load can have negative effects by decreasing the attention, precision, working memory, and effectiveness of the individual (Coursaris et al., 2012) which can in turn increase stress (Sanders and Baron, 1975). Previous studies on stress examined that stress induced by the use of technology affect user's psychological well-being (Ayyagari et al., 2011; Thomée et al., 2011; Choi and Lim, 2016).

Distraction is due to a lack of attention; the absence of interest in the topic; and the great intensity, novelty or attraction of something other than the object of interest (Craik, 2014). It comes from both internal and external sources (Nicholson et al., 2005). External distractions include factors like visual triggers, social interactions, music, text messages, and telephone calls. While internal distractions include hunger, tiredness, illness, anxiety, and daydreaming. The interference of focus is supported by both external and internal distractions (Schumm and Post, 1997). Distraction-conflict theory provides insight into the evaluation of social media as “other” technology that distracts people from their primary goal (Leung, 2015). Negative consequences of distraction include effort difficulties and mental attention (Baecker et al., 1995) and impaired task performance (Cellier and Eyrolle, 1992; Suh et al., 1996).

Concerning mobile phones, its ubiquity and easy access makes it a potentially strong mechanism for distraction (David et al., 2015). Mobile phone distractions can be initiated by sound (when a user gets a message or call) or by sight (when receiving a notification from social networking site posts, online notifications of friends and family available on social networking sites) (Brooks, 2015). Users wonder what their friends and family are doing on social networking sites, scrolling and commenting on friend and family moments, sending videos and pictures, playing games, watching videos, online shopping, and listening to music only to engage themselves in mobile phone activities (Wu et al., 2018). Therefore, the mobile phone has made distraction easier, due to their portability and the diversity of entertaining features. Even when users are doing work activities and studying (Thomée et al., 2011; Zhou, 2019), their primary focus is distracted by mobile phone technology (Coursaris et al., 2012). Therefore, the current study aims to test a proposed research model based on distraction-conflict theory to expand theoretical knowledge about whether and how mobile distraction, cognitive emotional pre-occupation and attention control affects users' psychological well-being.

### Mobile Phone Distraction and Cognitive Emotional Pre-occupation

The use of mobile phone technology can lead to sacrificing other goals such as neglecting other commitments and a decrease in social activities with friends and family (Lin, 2019). The increased use of mobile phone technology in the daily life developed user's checking habits whereby they constantly make a brief inspection of their mobile phone applications (Porter and Kakabadse, 2006; Yang et al., 2016). It diverts the user's attention to non-work-related activities (Ou and Davison, 2011; Rosen et al., 2013; Ziegler et al., 2018).

The diversity of mobile phone features and functions induce excessive usage behavior (Oulasvirta et al., 2012) and users experience difficulty in controlling the time they spend on the device and are easily distracted (Bianchi and Phillips, 2005). Such distraction stimulates cognitive emotional pre-occupation with behavior (King et al., 2013). Cognitive emotional pre-occupation is defined as “obsessive thought patterns involving technology use” (Caplan and High, 2006).

Pre-occupation with a behavior produces strong cravings to engage in the behavior which leads to problematic behavior (Collins and Lapp, 1992). Users with excessive usage behavior, develop a strong link in their long-term memory and their behavioral tendencies are associated with their reactions (Strack and Deutsch, 2004). The existing literature about addiction or pathologic use tends to consider cognitive emotional pre-occupation as one of the core symptoms of problematic technology use (Nicholson et al., 2005). Cognitive emotional pre-occupation with mobile phone technology creates a strong willingness to use, which a mobile user may find difficult to endure and therefore, can act as a source for unplanned and even problematic use of the mobile phone (Cao et al., 2018). With the use of a mobile phone, an increased level of pre-occupation develops strong thoughts and emotional attachments, and the users feel a powerful urge to use even in a dangerous situation, where it is banned such as when driving a vehicle (Telemaque and Madueke, 2015; Turel and Bechara, 2016). Therefore, we hypothesized that

*H1: Mobile phone distraction is positively related to cognitive emotional pre-occupation*.

The diverse features of mobile phones increase the cognitive demand of users to use it. Such cognitive demand causes cognitive distraction. Cognitive distraction is defined as the user's difficulty to process two or more types of information at the same time (David et al., 2015). Phone calls, texting, and social media networking sites may cause a lapse in attention and concentration.

Previous research found that on-going use of mobile phone technology causes psychological distress (Chesley, 2005; Błachnio et al., 2013). Users expect enjoyment from the utilization of mobile phone technology but the loss of control on mobile phone usage affects cognitive limits and induces negative emotions. Previous research studies have found that mobile phone usage is negatively related to the concept of well-being, mood and anxiety disorder, fatigue, and mental health symptoms such as depression and sleep disturbance (Thomée et al., 2007, 2011; Dhir et al., 2018; Lin, 2019). Therefore, we hypothesized that

*H2: Mobile phone distraction has a significant negative relationship with psychological well- being*.

### Cognitive Emotional Pre-occupation and Psychological Well-Being

Excessive use of a mobile phone leads to a reduction in the daily working routine, productivity, physical health, social relationships, and emotional well-being (Horwood and Anglim, 2018). A recent study explored how the excessive use of a mobile phone induces stress (Zheng and Lee, 2016). The continuous use, news and information, demands for attention from social networking sites, work activities and several forms of entertainment results in cognitive emotional pre-occupation (Lee et al., 2014). Cognitive emotional pre-occupation develops clusters in the long-term memory of the users (Strack and Deutsch, 2004). These clusters have strong impulses on behavior such as cognitive or emotional reactions (Craik, 2014). The pre-occupation can be disturbing because, in the presence of such pre-occupying ideas and feelings, individuals find it hard to concentrate on other tasks (Fillmore, 2001). These negative emotions weaken psychological well-being and eventually lead to disregarding essential elements of a user's life such as their family, education, and work (Choi and Lim, 2016). Therefore, we hypothesized that

*H3: Cognitive emotional pre-occupation is negatively related to psychological well-being*.

### Cognitive Emotional Pre-occupation as a Mediator

We expected that cognitive emotion pre-occupation performs a mediating role in the relationship of mobile phone distraction and psychological well-being for the following reasons. First, mobile phone distraction causes excessive use which generates emotional and cognitive pre-occupation with behaviors (Cao et al., 2018). Such behaviors cause a strong desire to use a mobile phone to develop, which is difficult to resist (Zheng and Lee, 2016). This increased use of the mobile phone causes strong thoughts and emotional attachment to develop, leading to depression, which ultimately causes their well-being to deteriorate (Lee et al., 2014; Zhou, 2019). Second, previous studies have conceptualized that mobile phone users are extensively pre-occupied or “addicted” and overwhelmed with information, which reduces their cognitive capacity to manage the information effectively (Eppler and Mengis, 2004). When the user's cognitive limit exceeds the optimum level of technology utilization it may result in negative consequences (Ahuja et al., 2007). A previous study showed that mobile phone usage is negatively related to the concept of well-being that leads to interpersonal problems (Griffiths, 2005). Third, a compulsive desire to use the mobile phone can result in negative emotions such as emotional exhaustion, fatigue, and anxiety which affects their health and social relationships (Merrill and Liang, 2019). Such emotions reduce the psychological well-being of the users (Dhir et al., 2018). Therefore, we hypothesized that

*H4: Cognitive emotional pre-occupation mediates the relationship of mobile phone distraction and psychological well-being*.

### Attention Control as a Moderator

Mobile phones increase people's enjoyment and comfort by providing them with flexible access to information which can turn into excessive use of the mobile phone (Yang et al., 2016). Such activities distract users from their routine work and enhances the cognitive and behavioral intentions of the users. However, due to attentional conflict, mobile phone distraction can have significant implications, ranging from short-term inconvenience (e.g., annoyance) to life-threatening circumstances such as motor accidents (Turel and Bechara, 2016). According to Ellis et al. (2014) and Hu et al. (2017) the ability to control attention switching and maintaining the negative affective response effect is known as attention control (AC). Some researchers suggest that individual differences in working memory capacity represent a different attention control on the use of working memory resources (Engle, 2002; Fukuda and Vogel, 2011). Attention control such as self- regulation ability, starting, maintaining concentration, and shifting internal and external attention to ensure flexibility is used to remain focused (Chambers et al., 2008). According to Derakshan and Eysenck (2009), attention control helps to increase processing efficiency and cognitive performance of an individual plays a critical role in decreasing data processing complexity and focusing on the concentrated goal. Therefore, this study uses attention control that helps to reduce the negative consequences of mobile phone distraction, because it may influence the capacity to neglect adverse cognitive and emotional consequences. Furthermore, evidence shows that distraction due to mobile phones has an impact on user's behavior (Craik, 2014). According to Cao et al. (2018) cognitive emotional pre-occupation produces problematic behavior which can affect psychological well-being. Moreover, attention control helps to reduce depressive disorder (Hu et al., 2017). Thus, the study suggested that mobile phone distraction influences user's cognitive emotional behavior and affects their psychological well-being. The indirect relationship weakens when users have high attention control. Therefore, we hypothesized that

*H5: Attention control moderates the effect of mobile phone distraction and cognitive emotional pre-occupation the weaker the relationship with high attention control*.
*H6: Attention control moderates the mediating effect of cognitive emotional pre-occupation between mobile phone distraction and psychological well-being*.

**Figure 1:**
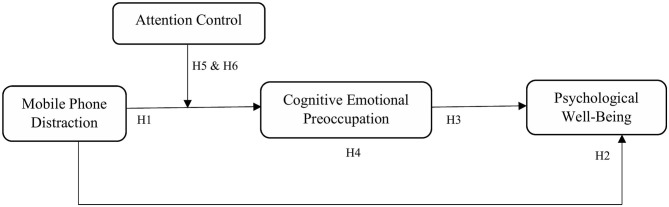
Proposed research model.

Figure 1 shows the proposed theoretical framework.

## Methodology

### Sample and Data Collection

To examine the reliability and validity of the construct, a pilot study was conducted before the data collection process. The questionnaire was distributed online to 50 volunteer students in a large University in China. We performed exploratory factor analysis to ensure the reliability and validity of the construct. On the basis of findings, two items were removed from the revised final questionnaire. Empirical data were collected online by sharing the link of the questionnaire amongst social groups of University students (WeChat, Weibo and QQ) and by sending invitations to students *via* University email. The targeted sample involved students from a large University in China. This sample is suitable considering that the younger generation make up the majority of active users as they constitute the main body of mobile phone users. Therefore, students are considered as an adequate source of data for this study. Compeau et al. (2012) validated that University students represent part of the population, and their characteristics are similar to population characteristics. According to Kuss et al. (2013), students are more prone than others to present problematic online technology usage behavior. To ensure the quality of records we asked students to fill their student ID number in questionnaire so that repetition and redundancy of records will be removed. All participants were assured that their data will remain confidential and that it was collected for research purposes only. A convenience sampling technique was used to collect the data. A back-translation method was employed because the original questionnaire items were developed in English. Thus, the items were translated into Chinese by a Chinese translator for the data collection process and were subsequently converted back into English for further analysis (Brislin, 1970). The sample size was calculated by using the Godden (2004) formula for an infinite population (recommended sample was 384). A total of 935 survey responses were collected. After outliers and incomplete responses were eliminated, a total of 914 responses were gathered for further analysis.

### Measures

We adapted the questionnaire from the literature and some items were modified according to the context of the current study. All items were measured using a 5-point Likert scale ranging from 1 = strongly agree to 5= strongly disagree. The measurement items of all variables were described in **Table 2**. The demographic variables such as age, gender, and frequency of use were measured as control variables.

### Mobile Phone Distraction

Mobile phone distraction was assessed using a four-items construct and it was adapted from Davis et al. (2002). The items represent the frequent use of a mobile phone while performing other activities. The Cronbach's alpha (CA) value is 0.95.

### Cognitive Emotional Pre-occupation

Cognitive emotional pre-occupation was measured using a six-items scale and was adapted from Caplan and High (2006) and Zheng and Lee (2016). The items represent the feeling of an urge and thoughts to use a mobile phone when not using it for some time. The CA value is 0.95.

### Psychological Well-Being

Psychological well-being was measured using an eight-items scale that was adapted from Steinfield et al. (2008) and Choi and Lim (2016). The items consist of positive and negative wording, negative items were reverse coded to measure the psychological well-being. The CA value is 0.96.

### Attention Control

An eight-items scale was adapted from Farmer and Sundberg (1986) and Brooks (2015) to measure attention control. The items represent frequent shifting of attention during distraction and focusing on the main task. Some items were reverse coded to measure the positive effects of attention control. The CA value is 0.97.

## Data Analysis and Results

For data analysis, we used IBM-SPSS 22, IBM-AMOS 23, and Process macro by Hayes.

First, we performed the descriptive analysis to measure the demographic data. The demographic data of 914 students were based on males (48.1%) and females (51.9%). The remaining demographic data of 914 respondents are given in Table 1. Second, we performed exploratory factor analysis to measure the reliability and validity of the constructs. Third, we performed structural equation modeling (SEM) using IBM- AMOS 23 to find out the confirmatory factor analysis and model fit indices. Finally, we used Process macro in IBM-SPSS 22 to perform moderated-mediation analysis.

**Table 1:** Descriptive statistics of respondent characteristics.

**Measure**	**Value**	**Frequency**	**Percentage (%)**
Gender	Male	440	48.1
	Female	474	51.9
Age (Years)	Below 18	2	0.2
	18–22	84	9.2
	23–27	266	29.1
	28–32	416	45.5
	33–37	128	14.0
	38–42	14	1.5
	Above 42	4	0.4
The frequency of mobile	Many times a day	504	55.1
phone use	Hourly	254	27.8
	Once a day	94	10.3
	Less than once a day	62	6.8

### Validity and Reliability of the Measurement Items

Reliability pertains to the consistency of the construct, and validity pertains to how the constructs define the concept of the study (Carmines and Zeller, 1979). This study performed the exploratory factor analysis (EFA) using a principle component analysis with varimax rotation and a suppressed value of <0.50 to measure the validity of the construct. The results of the principle component analysis produced four factors with an Eigen value >1 explaining 83.26 % of the total variance. All factor loadings on the expected factors are within the range of 0.81 to 0.93 (see Table 2) while the recommended values should exceed 0.7 to ensure construct validity (Hair et al., 1998). To measure the reliability of the constructs, we used CA and composite reliability (CR) values. The values of CA and CR must exceed the threshold of 0.7 (Anderson and Gerbing, 1988). Table 2 indicates that all CA and CR values exceed 0.7, thereby ensuring measurement reliability. We also checked the average variance extracted (AVE) for convergent validity. In our data, the average variance extracted values of constructs ranged from 0.74 to 0.80, greater than the minimum threshold of 0.5 as recommended by Fornell and Larcker (1981) which indicates that the items satisfied the convergent validity requirements.

**Table 2:** Confirmatory factor analysis, AVE and composite reliability.

**Variables**		**Items**	**Factor loading**	**Cronbach's alpha**	**AVE**	***CR***
Mobile phone distraction	MD1	When I am using a mobile phone, I don't think about my work/tasks	0.89			
	MD2	I lose track of time when using the mobile phone	0.87			
	MD3	I often use a mobile phone to avoid doing unpleasant things in my work/task	0.87	0.95	0.76	0.92
	MD4	I find that I use mobile phone when I have work to do	0.87			
Cognitive emotion pre-occupation	CG1	When I haven't been using mobile phone for some time, I become pre-occupied with the thought of using it	0.84			
	CG2	I would feel lost if I was unable to use mobile phone	0.80			
	CG3	I think obsessively about using mobile phone applications when I am not using them	0.88	0.95	0.75	0.94
	CG4	Do you find yourself unable to stop thinking about using mobile phone?	0.89			
	CG5	Is it hard to distract yourself from thinking about mobile phone?	0.90			
	CG6	Do thoughts about using mobile phone intrude into your daily activities?	0.89			
Attention control	AT1	When concentrating, can focus and become unaware	0.88			
	AT2	It is easy for me to read or write while I am also talking on phone	0.87			
	AT3	After being interrupted/distracted, easily shift attention back	0.90			
	AT4	When trying to focus my attention, I have difficulty blocking out distracting thoughts	0.90	0.97	0.80	0.96
	AT5	When I need to concentrate, I have trouble focusing my attention	0.93			
	AT6	When working on something, still get distracted by mobile phone	0.89			
	AT7	Distracting thought comes to mind, easy for me to shift my attention away from it	0.90			
	AT8	When I am reading or studying, I am easily distracted if notifications appear on mobile phone	0.89			
Psychological well-being	PW1	The demands of everyday life often get me down	0.84	0.96	0.74	0.95
	PW2	I am quite good at managing the many responsibilities of my daily life	0.81			
	PW3	I have difficulty arranging my life in a way that is satisfying to me	0.84			
	PW4	I don't have a good sense of what it is that I am trying to accomplish in my life	0.90			
	PW5	In general, I feel confident and positive about myself	0.92			
	PW6	In many ways, I feel disappointed about my achievements in my life	0.82			
	PW7	My attitude about myself is probably not as positive as most people feel about themselves.	0.86			
	PW8	I am able to do things as well as most other people	0.89			

Discriminant validity is the square root of all AVE values greater than the off-diagonal correlations between the constructs. Table 3 shows that the value of the square root of AVE is greater than the correlation coefficient of the constructs, thereby indicating discriminant validity.

**Table 3:** Correlations, mean, and standard deviation.

**Sr. no**	**Variables**	**Mean**	**SD**	**1**	**2**	**3**	**4**
1	Mobile phone distraction (MD)	3.8	1.018	**(0.87)**			
2	Cognitive emotion pre-occupation (CG)	4.1	0.772	0.298^**^	**(0.86)**		
3	Attention control (AT)	2.1	0.887	−0.472^**^	−0.171^**^	**(0.89)**	
4	Psychological well-being (PW)	4.1	0.834	0.234^**^	0.450^**^	−0.173^**^	**(0.86)**

The diagonal elements (in bold) are the square root of variance shared between the AVE, whereas the off-diagonal elements are the correlations among constructs. ^*^p < 0.05;

** *p < 0.01*;

^***^p < 0.001.

Furthermore, we used IBM AMOS 23 to conduct confirmatory factor analysis (CFA) for validating the measures. The value of CMIN/*df* = 2.75, NFI = 0.98, TLI = 0.96, IFI = 0.98, CFI = 0.97, RMSEA = 0.05 indicated a valid model fit. The results indicated that the values are within the acceptable range as suggested by Hair et al. (1998). Therefore, the results show a valid model fit.

We performed the Harman's one-factor test to evaluate the extent of common method bias (Podsakoff et al., 2003) because all questions were answered by the same individual. In this test, the threat of common method bias is considered high if a single factor account for more than 50% of total variance (Harman, 1976). The results reveal that none of the factors dominate the explanation of the variance, in which the most influential factor accounts for 36.9% of the variance. Moreover, other evidence of a common method bias includes high correlations (*r* > 0.9) among variables (Pavlou and El Sawy, 2006). Table 3 shows that unusually high correlation in the sample is non-existing.

Thus, the common method bias is not a serious concern in this study.

### Structural Model

Structural equation modeling (SEM) was used to measure the model fit indices. The results of model fit indices show that the model was a good fit [χ^2^ (666.752), *df* = 248, χ^2^/*df* =2.68, NFI = 0.96, IFI = 0.97, CFI = 0.97 and RMSEA = 0.06]. The proposed model is within the acceptable range that is defined by Anderson and Gerbing (1988); in particular, χ^2^/*df* < 5, NFI > 0.90, IFI > 0.90, CFI > 0.90 and RMSEA < 1.0.

### Hypothesis Testing

**Figure 2:**
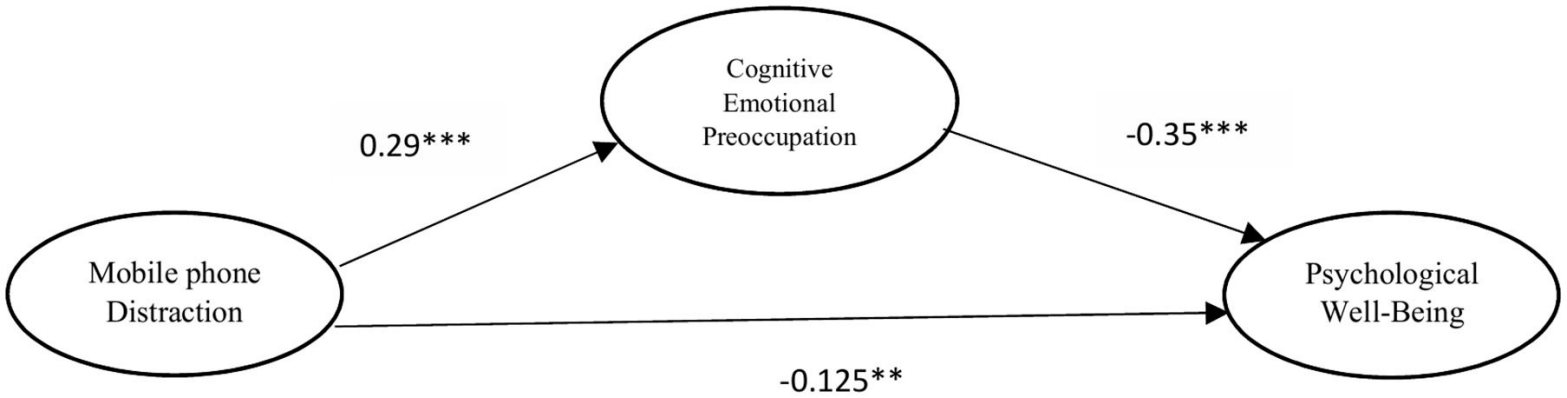
SEM path diagram.

This study used structural equation modeling to test the direct and mediation hypothesis. The results of direct and indirect effects are given in Table 4. The relationship between mobile phone distraction and cognitive emotional pre-occupation (β = 0.29, *p* < 0.001) was significant, leading to the acceptance of hypothesis 1. The results indicate that the direct effect of mobile phone distraction and psychological well-being (without mediator) is significant (β = −0.23, *p* < 0.001), leading to the acceptance of hypothesis 2. The relationship between the cognitive emotional pre-occupation and psychological well-being (β = −0.35, *p* < 0.001) was also significant, indicating the acceptance of hypothesis 3. The path diagram of SEM is demonstrated in Figure 2. We used the bootstrapping method with 5,000 bootstrap samples and a 95% confidence interval for indirect effect. The bootstrapping result of the indirect effect of mobile phone distraction on psychological well-being *via* cognitive emotional pre-occupation is also significant (β = −0.08, *p* < 0.01). Hence, cognitive emotional pre-occupation partially mediates the relationship between mobile phone distraction and psychological well-being, thereby accepting hypothesis 4. Regarding weak beta coefficient, previous studies have also identified weak beta value of indirect effect (Qian et al., 2017; Liu and Li, 2018). Furthermore, we used ANOVA to check the significant differences of control variables (gender, age, and frequency to use). The control variables exhibit insignificant effects on psychological well-being. Therefore, we exclude the control variables for further analysis.

**Table 4:** Bootstrap results for direct and indirect effects.

**Path (direct effect)**	**Estimates**		**SE**	
MD→ PY (without mediator)	−0.23^***^		0.03	
MD→ CG	0.29^***^		0.03	
CG→ PY	−0.35^***^		0.04	
MD→ PY (with mediator)	−0.125^**^		0.03	
**Path**	**Effect**	**SE**	**LL 95% CI**	**UL 95% CI**
**Indirect effect (bias corrected confidence interval method)**				
MD→ CG→ PY	−0.08^**^	0.021	−0.134	−0.050

** *p < 0.01*,

*** *p < 0.001*,

*MD, Mobile phone distraction; PY, psychological well-being; CG, cognitive emotional pre-occupation; LL, lower limit confidence interval; UL, upper limit confidence interval; SE, standard error*.

### Moderated Mediation Analysis

**Figure 3:**
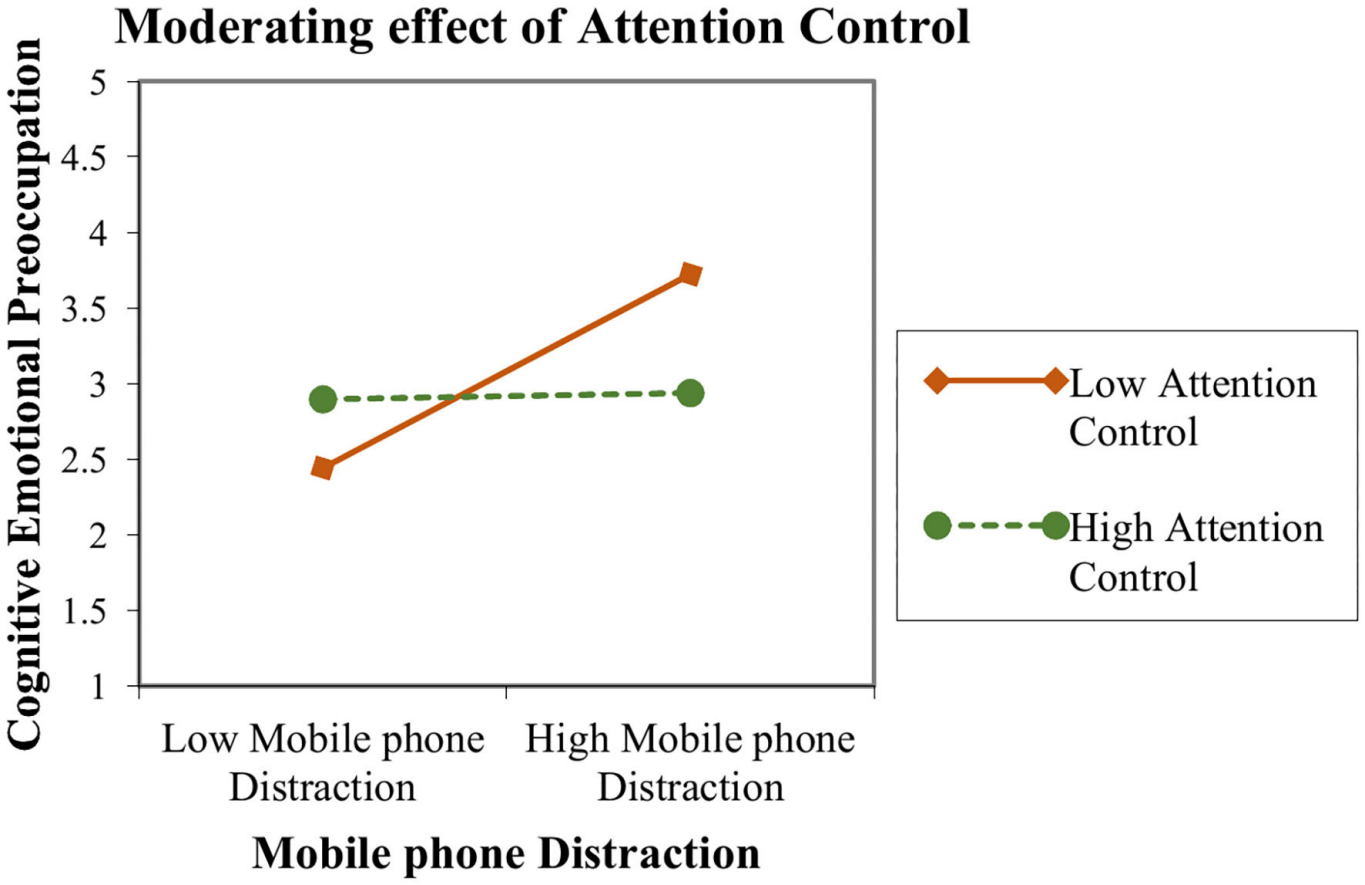
Moderating effect of attention control between the relationship of mobile phone distraction and cognitive emotional pre-occupation.

The moderated-mediation results are described in Table 5. The current study hypothesized a moderating role of attention control between mobile phone distraction and cognitive emotional pre-occupation. We used model 7 of the Process macro by Hayes (2013) in IBM-SPSS 22 to analyze the moderated mediation analysis. Interestingly, the results showed that the relationship between mobile phone distraction and cognitive emotional pre-occupation is highly significant when attention control is low (β = 0.44, *p* < 0.001) but becomes weakest and insignificant when attention control is high (β = 0.05, *p* > 0.05). Figure 3 shows the graphical presentation of moderating effect of attention control which describe that slop is becoming less positive as move from low to high attention control. Therefore, hypothesis 5 was supported and accepted.

**Table 5:** Moderated mediation model of attention control, mobile phone distraction, cognitive emotional pre-occupation, and psychological well-being (model 7 process macro, *n* = 914).

	**Cognitive**		**Psychological**	
	**emotional pre-occupation**		**well-being**	
	**β**	**SE**	**β**	**SE**
**Explained variables**				
MD	0.25^***^	0.26	0.06^***^	0.01
AT	−0.07^**^	0.29		
CG			0.301^***^	0.02
MD × AT	−0.222^***^	0.02		
**Levels of AT**	**β**	**SE**	**95 % CI**	
			**LL**	**UL**
High (+1 *SD*)	0.447	0.03	0.3792	0.5166
Moderate	0.2511	0.02	0.1998	0.3024
Low (−1 *SD*)	0.0543	0.03	−0.0051	0.1136
**Index of moderated mediation**				
	index = −0.0711	SE = 0.0103	CI = [−0.0927, −0.0516]	

*MD, mobile phone distraction; AT, attention control; CG, cognitive emotional pre-occupation; CI, confidence interval; LL, lower limit; UL, upper limit*.

** *p < 0.01*,

*** *p < 0.001*.

Furthermore, the conditional indirect effect is reflected in the index of a moderated mediation analysis and if zero does not fall between the lower and upper limit of the 95% confidence interval then the indirect effect is conditional on the level of the moderator (Preacher and Hayes, 2008). The result of moderated mediation analysis [index = −0.0711, SE = 0.0103, CI = (−0.0927, −0.0516)] shows that attention control fully moderated the mediation effect of cognitive emotion pre-occupation between mobile phone distraction and psychological well-being, thereby accepting hypothesis 6.

## Discussion

### Findings

On the basis of distraction conflict theory, the current study proposed a research model to examine the effects of mobile phone distraction on psychological well-being. Specifically, the current study has the following aims. First, to examine the effect of mobile phone distraction and cognitive emotional pre-occupation. Second, to investigate the impact of mobile phone distraction on psychological well-being. Third, to examine the mediating effect of cognitive emotional pre-occupation between mobile phone distraction and psychological well-being.

Finally, to study how attention control moderates the mediation effect of cognitive emotional pre-occupation between mobile phone distraction and psychological well-being. The key contribution of the current study is to examine mobile phone distraction in relation to attention control, cognitive emotional pre-occupation, and psychological well-being in China. The findings of the study support the proposed model and hypotheses and provides important theoretical and practical implications.

The current study resulted in several important findings. First, the current study contributes to the mobile phone distraction literature by identifying its consequences. Our result shows that mobile phone distraction exhibits a positive and significant relationship with cognitive emotional pre-occupation. Users have strong willingness to use mobile phone technology which causes strong emotional attachments to develop and users feel a powerful urge to use their mobile phone. Excessive use of mobile phone SNS is positively associated with cognitive emotional pre-occupation of Chinese students (Cao et al., 2018). Similarly, Coursaris et al. (2012) and Longstreet and Brooks (2017) found that mobile phone distraction has an impact on the efficiency and effectiveness of users which in turn influence user's satisfaction and behavioral intention toward the usage of mobile phones. Moreover, Alalwan et al. (2018) and Leung (2015) revealed that mobile phone distraction has a positive relationship with perceived enjoyment and task performance.

Second, the excessive use of a mobile phone can result in lower psychological well-being. Our findings revealed that mobile phone distraction has a negative and significant association with psychological well-being. So much so that mobile phone usage limits the cognitive ability of the user so that they are not be able to focus on daily routine activities which leads to negative psychological well-being. Taiwanese students highly depend on mobile phone usage and perceive that being permanently connected to a mobile phone causes stress (Lin, 2019). Turel and Bechara (2016) found that mobile phone usage during driving distract users which ultimately has negative outcomes (e.g., accidents). Similarly, Schwebel et al. (2012) found that mobile phone distraction (e.g., talking on the phone, texting, and listening to music) has a negative impact on pedestrian behaviors.

Third, many scholars have found that an increase in use of a mobile phone can result in psychological consequences (e.g., anxiety, depression, fatigue, exhaustion) (Bianchi and Phillips, 2005; Thomée et al., 2011; Zheng and Lee, 2016). Excessive use of a mobile phone is positively related to mobile phone addiction, exerting a direct impact on psychological well-being in young Korean adults (Choi and Lim, 2016; Cha and Seo, 2018). Tangmunkongvorakul et al. (2019) shows that excessive use of a mobile phone has a negative effect on user's psychological well-being.

Similarly, Sahin and Çoklar (2009) and Dhir et al. (2018) found that compulsive use of a mobile phone increases fatigue and stress levels, and ultimately effects users' psychological well-being. Moreover, Cao et al. (2018) found that excessive use of a mobile phone causes cognitive-emotional pre-occupation which in turn has a positive relationship with psychological strains (e.g., life invasion, techno-exhaustion, and privacy invasion). Our findings show that cognitive emotional pre-occupation has a negative and significant relationship with psychological well-being. This study predicts that the concentration demand of social networking sites, text messages, calls, and other mobile features grab user attention, influencing their negative emotional reactions and behaviors and ultimately lowering users' psychological well-being.

Fourth, our findings show that cognitive emotional pre-occupation partially mediate the relationship of mobile phone distraction and psychological well-being. Higher mobile phone use is associated with lower well-being (Volkmer, 2019). Similarly, users with high levels of cognitive emotional pre-occupation with the internet will experience more negative outcomes (Caplan and High, 2006). Therefore, users who spend more time online are more likely to exhibit an increase in depression and social separation.

Finally, the study identified an important variable—attention control—which helps users to cope with the negative impact of mobile phone distraction and help to avoid getting emotionally connected. Our finding indicates that users with low attention control, experience more cognitive attachment and face attention conflicts with the mobile phone, whereas users with high attention control do not experience such an attachment and are more focused on their goals. The results are in line with the study of Hu et al. (2017) who suggested that attention control helps to reduce depressive disorder. Moreover, Derakshan and Eysenck (2009) and Jung et al. (2019) found that attention control helps to increase cognitive performance and efficiency, and improves the decision making process of individuals.

### Theoretical and Practical Implications

This study exhibits certain important theoretical implications. First, the current study contributes to the existing literature on mobile phone distraction by examining the underlying mechanism through which mobile phone distraction affects psychological well-being. The study theoretically expands the etiology of problematic mobile phone use and discusses its potential adverse effect. The current study extends the literature on distraction-conflict theory by emphasizing that mobile phone distraction negatively affects psychological well-being. It also validates the distraction conflict theory by examining its validity on the mobile phone distraction and cognitive emotional behavior. Cognitive emotional pre-occupation is a new phenomenon in the field of mobile phone distraction. Second, the current research aims to enhance the understanding association of mobile phone distraction with cognitive emotion pre-occupation and its impact on psychological well- being. Finally, the study complements previous studies on attention control and contributes to the literature by examining the moderating effect of attention control in the association between mobile phone distraction, cognitive emotional pre-occupation, and psychological well-being which previous studies have not examined.

The current study has some practical implications. First, to avoid the negative consequences of mobile phone distractions, users must reduce their usage and manage their behaviors accordingly to overcome psychological issues. Second, the findings also have implications on policies where institutions must educate students about the negative psychological consequences of excessive use of a mobile phone so that they can reduce their usage while performing their routine work. Finally, this study also suggests that users with high attention control are not affected by the negative consequences of mobile phone distraction. Therefore, users should be more focused on their goals and limit the usage of a mobile phone to avoid negative consequences.

### Limitation and Future Research

The current study had certain limitations. First, data was collected from University students which was the best fit for our research study. It is an empirical question as to whether the findings can be generalized to other countries and cultures. Various cultural factors, values, and beliefs have an impact of individual psychological well-being (Wissing and Temane, 2008; Grossi et al., 2012). Future research must focus on different target samples in other work settings or be conducted in a cross-cultural study of different countries to elucidate more interesting results. Particularly, researchers should focus on cultural factors such as gender, education and occupation to examine the effect of mobile phone distraction on psychological well-being. Second, the study focused on overall mobile phone distraction and was not specific to any mobile application such as social networking sites applications, mobile-gaming applications, etc. Future research must be focused on distraction caused by these applications to examine its effects on users' behavioral intentions. Third, the current study used control variables e.g., age, gender, and frequency of use, therefore, future research should use other control variables such as time and experience to find out more interesting results. Finally, the study considered the users' psychological well-being rather than focusing on specific psychological factors. Further investigation should extend this study to explore each factor of psychological well-being such as anxiety, sleep disorder and exhaustion, and should also examine its effect on physical and emotional well-being.

### Conclusion

The current study was primarily focused on the implications of mobile phone distraction on psychological well-being. This study's greatest contribution was the finding that mobile phone distraction stimulates cognitive emotional pre-occupation with behavior and undermined user's psychological well-being. Moreover, users with high attention control, can easily manage their daily routine activities and ensure flexibility to remain focused. If the different factors proposed in the limitation of this study are included in future research, they could provide more interesting results of the negative functions of mobile phone usage.

## Data Availability Statement

The raw data supporting the conclusions of this article will be made available by the authors, without undue reservation.

## Ethics Statement

Ethical review and approval was not required for the study on human participants in accordance with the local legislation and institutional requirements. Written informed consent from the participants was not required to participate in this study in accordance with the national legislation and the institutional requirements.

## Author Contributions

All authors listed have made a substantial, direct and intellectual contribution to the work, and approved it for publication.

## Conflict of Interest

The authors declare that the research was conducted in the absence of any commercial or financial relationships that could be construed as a potential conflict of interest.
